# Relation between iodine nutrition and thyroid diseases in Qinghai, China

**DOI:** 10.3389/fendo.2023.1234482

**Published:** 2023-09-08

**Authors:** Xiaoxia Fan, Lingling Zhao, Shuqiong Wang, Kang Song, Beibei Wang, Yanling Xie, Yanping Jiang, Lijun Lin, Weiping Teng, Chunmei Cai, Yongli Yao

**Affiliations:** ^1^Department of Endocrinology, Qinghai Provincial People’s Hospital, Xining, China; ^2^Key Laboratory of Ministry of Education for High Altitude Medicine, Research Center for High Altitude Medicine, Xining, China; ^3^Key Laboratory of Application and Foundation for High Altitude Medicine Research in Qinghai Province (Qinghai−Utah Joint Research Key Lab for High Altitude Medicine) Qinghai University, Xining, China; ^4^Department of Endocrinology and Metabolism, Institute of Endocrinology, The First Affiliated Hospital of China Medical University, Shenyang, China

**Keywords:** Qinghai province, thyroid disease, iodine nutrition, epidemiology, relation

## Abstract

**Objective:**

To investigate the adult iodine nutrition and the prevalence of thyroid diseases in Qinghai Province, and analyze the correlation between iodine and thyroid diseases, so as to provide a basis for adjusting the salt iodization plan in Qinghai Province.

**Methods:**

Using cluster and stratified sampling method to select 2628 permanent residents over 18 years old in Qinghai Province for questionnaire survey, physical examination, thyroid color ultrasound, and laboratory index detection.

**Results:**

1. The coverage of iodized salt in adults is 99.71%. 2. The detection rates of thyroid disorders in adults were as follows: Clinical hyperthyroidism was 1.20%, subclinical hyperthyroidism was 0.20%, clinical hypothyroidism was 1.00%, subclinical hypothyroidism was 29.20%, and the goiter was 2.10%. The percentages positivity of TPO Ab, TG Ab, goiter was 9.80%, 9.20%, 2.10%, respectively. Among them single thyroid nodule was 6.40%, multi-nodule thyroid gland was 1.80%. 3. The percentages of mild iodine deficiency, moderate iodine deficiency, Severe iodine deficiency, adequate iodine intake (AI), more than adequate iodine intake (MAI)and excessive iodine intake (EI)were 8.41%, 2.17%, 0.26%, 33.22%, 28.35%, and 27.59%, respectively. The percentages of mild, moderate and severe iodine deficiency in urban populations (7.13%, 0.87%, 0.0%) were significantly lower than those in rural populations (9.81%, 3.59%, 0.56%) (P < 0.05), and the rates of adequate, more than adequate iodine intake in urban populations (36.03%, 30.93%) were significantly higher than that in rural populations (30.14%, 25.52%). The rate of excess iodine intake was higher in rural areas (30.38%) than in urban areas (25.04%). 4. The positive rates of subclinical hypothyroidism, goiter, TPO Ab and TG Ab in female adults (35.28%, 3.39%, 13.54%, 13.94%) were higher than those in male adults (23.58%, 0.96%, 6.266%, 4.79%). The detection rate of single thyroid nodules was higher in urban (8.01%) than rural populations (4.70%), while the detection rate of hypothyroidism, subclinical hypothyroidism, and goiter (0.58%, 25.84%, 1.38%) was lower than that in rural populations (1.52%, 32.96%, 2.96%) (P<0.05). 5. There was no statistical significance in the detection rates of clinical hyperthyroidism, subclinical hypothyroidism, subclinical hypothyroidism, goiter, thyroid nodules, TPO Ab and TG Ab positive rates in different iodine nutritional status (P>0.05). The positive rate of hypothyroidism in the iodine deficiency group is higher than in other iodine nutrition groups.

**Conclusion:**

The nutritional status of iodine in Qinghai Province is iodine excess. Subclinical hypothyroidism was detected at a high rate. Subclinical hypothyroidism, goiter, TPO Ab, and TG Ab were more common in female than in male. The proportion of mild, moderate, and severe iodine deficiency was higher in urban areas than in rural areas. The detection rate of thyroid nodules was higher in urban than in rural areas, and that of hypothyroidism, subclinical hypothyroidism, and goiter was lower than that in rural populations. The detection rate of clinical hypothyroidism was statistically significant in different iodine nutritional states (P< 0.05).

## 1 Highlights

- The first large-scale investigation of the association of iodine nutritional status with thyroid disease was reported in Qinghai Province since China implemented Universal Salt Iodization (USI) legislation.
- 2650 individuals were surveyed, of whom 2628 had completed data. Iodized salt coverage in adults was 99.71%. The median urinary iodine level of the adult populations was 217.90 µg/L.
- The detection rates of thyroid disorders in adults were as follows: Clinical hyperthyroidism was 1.20%, subclinical hyperthyroidism was 0.20%, clinical hypothyroidism was 1.00%, subclinical hypothyroidism was 29.20%, and the goiter was 2.10%. The percentages positivity of TPO Ab, TG Ab, goiter was 9.80%, 9.20%, 2.10%, respectively. Among them single thyroid nodule was 6.40%, multi-nodule was 1.80%.
- The percentages of mild iodine deficiency, moderate iodine deficiency, severe iodine deficiency, adequate iodine intake (AI), more than adequate iodine intake (MAI)and excessive iodine intake (EI)were 8.41%, 2.17%, 0.26%, 33.22%, 28.35%, and 27.59%, respectively. The percentages of mild, moderate and severe iodine deficiency in urban populations (7.13%, 0.87%, 0.0%) were significantly lower than those in rural populations (9.81%, 3.59%, 0.56%) (P < 0.05), and the rate of adequate, more than adequate iodine intake in urban populations (36.03%, 30.93%) was significantly higher than that in rural populations (30.14%, 25.52%). The rate of excess iodine intake was higher in rural areas (30.38%) than in urban populations (25.04%).
- The positive rates of subclinical hypothyroidism, goiter, TPO Ab and TG Ab in female adults (35.28%, 3.39%, 13.54%, 13.94%) were higher than those in male adults (23.58%, 0.96%, 6.266%, 4.79%). The detection rate of single thyroid nodules was higher in urban (8.01%) than rural populations (4.70%), while the detection rate of hypothyroidism, subclinical hypothyroidism, and goiter (0.58%, 25.84%, 1.38%) was lower than that in rural populations (1.52%, 32.96%, 2.96%).
- No statistical significance in the detection rate of clinical hyperthyroidism, subclinical hyperthyroidism, clinical hypothyroidism, subclinical hypothyroidism, goiter, thyroid nodule, and TPO Ab positive ratio of different iodine nutrition conditions (P > 0.05). The detection rate of clinical hypothyroidism was statistically significant in different iodine nutritional states (P< 0.05).

## 2 Introduction

Thyroid disease is a global public health problem, and iodine nutritional status is closely related to thyroid disease (1). There was once a mild to moderate iodine deficiency in China until 1996, when the Universal Salt Iodization (USI) policy was implemented. In 2010, 28 provinces had eliminated iodine deficiency disorders. Iodine plays a key role in thyroid hormone synthesis and thyroid cell function. Insufficient or excessive iodine intake can cause thyroid problems (2). Iodine deficiency disease is one of the most serious epidemic diseases in Qinghai Province which is located in the northeastern part of the Qinghai-Tibet Plateau at an altitude of 1644 m to 6851 m. All 43 counties (cities, districts) in the province are severely iodine deficient areas. Through the government mandatory implemented of universal salt iodization, the coverage of iodized salt in our province has significantly improved, and the iodine nutrition status of the population has effectively improved. The national food safety standard “Iodine Content in Edible Salt” (GB 26878-2011) was adopted in 2012. Qinghai Province selected a salt iodization level of (30 ± 9) mg/kg according to the level of iodine nutrition of the population and the need to continuously eliminate the hazards of iodine deficiency. According to the 2016 survey, the urine iodine level of children aged 8-10 in Qinghai Province is appropriate. The TIDE program (3) found that Qinghai Province had changed from iodine deficiency to current iodine excess areas. However, there has been no report on the large-scale thyroid disease prevalence and associated thyroid disease relationship in Qinghai Province after USI, and the effect of iodine on the relationship with thyroid disease. This study investigated the iodine nutritional status, the changes in the prevalence and spectrum of thyroid diseases, and the impact of the relationship between iodine nutritional levels and thyroid disease in Qinghai Province, providing a scientific basis for determining the optimal iodine intake and reducing the risk of thyroid diseases for Qinghai people.

## 3 Materials and methods

### 3.1 Study participants

Inclusion criteria: Community residents aged>18 years, permanent residents who have lived in the region for more than 5 years. Exclusion criteria: pregnancy; severe liver and kidney disease; recent use of hormone drugs (except thyroid hormone replacement therapy). All participants provided written informed consent after receiving a thorough explanation of the research procedures.

### 3.2 Research method

#### 3.2.1 Sampling method

Cluster sampling was used to conduct a cross-sectional survey of permanent residents over 18 years of age in Qinghai province from September 2015 to March 2016. A total of 2650 people were surveyed, including 2628 with complete data. The participants of each age group are shown in **Table 1**. Among them, there are 1374 urban residents and 1254 rural residents.

**Table 1:** Age, gender, and urban-rural structure of the survey cohort.

Age (years)	Total(%)	Urban (n=1374)		Rural (n=1254)	
		Male	Female	Male	Female
18-29	636(24.20)	184	171	145	136
30-39	547(20.81)	148	135	125	139
40-49	568(21.61)	157	142	135	134
50-59	424(16.13)	108	95	127	94
60-69	263(10.01)	69	69	65	60
≥70	190(7.23)	45	51	50	44
Total	2628(100)	711	663	647	607

*Proportion of each age group: according to the standardized proportion of the 2010 national population census.

##### 3.2.1.1 Thyroid ultrasound examination

Thyroid ultrasound was performed using the US LOGIQ 100 PRO, GE, Milwaukee, Wisconsin, with a transducer probe frequency of 7.5 MHz.

##### 3.2.1.2 Thyroid function

Fasting venous blood samples from all investigated residents were collected. The central laboratory in Shenyang measured FT3, FT4, TSH, TPO Ab, and TG Ab by chemiluminescence immunoassay using a Cobas 601 analyzer (Roche Diagnostics, Switzerland).

##### 3.2.1.3 Urine iodine level detection

Collect the fasting urine of the subjects and use the urine sample to detect their iodine content. Urine iodine was measured by inductively coupled plasma mass spectrometry (Agilent 7700x, Agilent Technologies, USA).

#### 3.2.2 Definition of disease

According to the WHO/UNICEF/ICC IDD recommendations and the epidemiological study of thyroid diseases mentioned above, the classification of urinary iodine and diagnostic criteria for thyroid diseases (4, 5) are shown in **Table 2**.

**Table 2:** Diagnostic criteria for thyroid diseases and classification criteria for urinary iodine.

Thyroid disease	Diagnostic criteria
Clinical hyperthyroidism	TSH<0.27mIU/L;FT4>22pmol/L or FT3>6.8pmol/L
Subclinical hyperthyroidism (SH)	TSH<0.27mIU/L;FT4 versus FT3 within reference range(FT4 12.0-22.0pmol/L;FT3 3.1-6.8pmol/L)
Clinical hypothyroidism	TSH>4.2mIU/L;FT4<12pmol/L
Subclinical hypothyroidism (SCH)	TSH>4.2mIU/L;FT4 12-22pmol/L
TPO Ab positivity	TPO Ab>34IU/ml
TG Ab positivity	TG Ab>115IU/ml
Goiter	male>22.5ml;female >25.4ml
Thyroid nodule	At least one thyroid nodule in patients with non- thyroid enlargement(>5mm)
deficient iodine intake (DI)	Median urinary iodine <100µg/L
Mild iodine deficiency	Median urinary iodine 50-99µg/L
Moderate iodine deficiency	Median urinary iodine 20-49µg/L
Severe iodine deficiency	Median urinary iodine <20µg/L
Adequate iodine intake(AI)	Median urinary iodine 100- <200µg/L
More than adequate iodine intake (MAI)	Median urinary iodine 200- <300µg/L
Excessive iodine intake(EI)	Median urinary iodine ≥300µg/L

#### 3.2.3 Diagnostic criteria

The normal reference value range of each index in the laboratory examination is within the normal range of the applied reagent kit.

### 3.3 Statistical analysis

The data were statistically analyzed using SPSS26.0 software. The measurement data were represented by the median (quartile) [M (Q1, Q3)]. The comparison was carried out using the Z test and the comparison of multiple samples was carried out using the F test; X^2^ test or Fisher’s exact probability method was used to compare counting data; The difference was statistically significant with P<0.05.

## 4 Research results

### 4.1 General situation of survey objects

A total of 2650 participants completed the study, of which 22 were excluded due to lack of gender, age, or thyroid function testing information. Therefore, we analyzed the remaining 2628, 1374 in urban areas and 1254 in rural areas, including 1358 males (51.7%) and 1270 females (48.3%), with a balanced gender composition.

### 4.2 Results of the urine iodine test and iodized salt coverage rate in adults

Iodized salt is covered by 99.71% of adults (**Table 3**). The median population (urinary iodine, UIC) was 217.90 (148.08, 312.73) ug/L, the median UIC for male was 220.84 (153.97,315.51) ug/L and the median UIC for female was 213.87 (142.53, 310.79) ug/L. UIC did not differ significantly between adults of different sexes or ages (P>0.05)

**Table 3:** Comparison of UIC among adults of different sexes and ages [M (Q1, Q3), ug/l].

Variables	Number	UIC (ug/L)	Z/F	*P*
sex			1.34	0.521
Male	1358	220.84(153.97, 315.51)		
Female	1270	213.87(142.53, 310.79)		
Age/year			1.68	0.139
18-29	636	270.29(177.26, 484.92)		
30-39	547	225.96(151.10, 312.06)		
40-49	568	214.04(145.62, 304.36)		
50-59	424	200.70(140.35, 280.45)		
60-69	263	188.65(125.61,268.87)		
≥70	190	164.61(114.53,237.25)		

### 4.3 Comparison of iodine nutrition status among adults and different genders and regions

The percentages of mild iodine deficiency, moderate iodine deficiency, severe iodine deficiency, adequate iodine intake(AI), more than adequate iodine intake(MAI)and excessive iodine intake(EI)were8.41%, 2.17%, 0.26%, 33.22%, 28.35%, and27.59%, respectively. The percentages of mild, moderate and severe iodine deficiency in urban populations (7.13%, 0.87%, 0.0%) were significantly lower than those in rural populations (9.81%, 3.59%, 0.56%) (P < 0.05), and the rate of adequate, more than adequate iodine intake in urban populations (36.03%, 30.93%) were significantly higher than that in rural populations (30.14%, 25.52%). The rate of excess iodine intake was higher in rural populations (30.38%) than in urban populations (25.04%). Female and male had similar nutritional status (P>0.05) (**Table 4**).

**Table 4:** Comparison of adult iodine nutrition and iodine nutrition by gender and population.

Iodine state	N	Male (n=1358)		Female (n=1270)		χ^2^	*P*	Urban (n=1374)		Rural (n=1254)		χ^2^	*P*
		n	Percentage/%	n	Percentage/%			n	Percentage/%	n	Percentage/%		
Mild iodine deficiency	221	105	7.73	116	9.13	1.68	0.195	98	7.13	123	9.81	6.23	0.013
Moderate iodine deficiency	57	26	1.91	31	2.44	0.86	0.354	12	0.87	45	3.59	22.91	0.000
Severe iodine deficiency	7	4	0.29	3	0.24	0.08	0.772	0	0.0	7	0.56	7.71	0.005
Adequate iodine intake(AI)	872	440	32.40	432	34.02	0.87	0.35	495	36.03	379	30.22	11.83	0.001
More than adequate iodine intake(MAI)	745	400	29.46	345	27.17	1.40	0.237	425	30.93	320	25.52	9.01	0.003
Excessive iodine intake(EI)	725	383	28.20	342	26.93	0.53	0.468	344	25.04	381	30.38	9.74	0.002

### 4.4 Detection of thyroid diseases in adults

Thyroid diseases are detected in residents based on biochemical indicators such as TSH, FT3, and FT4, as the well as Doppler ultrasound color of the thyroid. The prevalence of thyroid disorders in adults were as follows: 1.20% of clinical hyperthyroidism, 0.2% of subclinical hyperthyroidism, 1.00% of clinical hypothyroidism, 29.20% of subclinical hypothyroidism, 9.80% of positive TPO Ab, 9.20% of positive TG Ab, 2.10% of goiter, 6.40% of single thyroid nodules and 1.80% of multiple nodules. The detection rate of subclinical hypothyroidism is as high as 29.20% (**Table 5**).

**Table 5:** Detection of thyroid diseases in adults of different sexes and population.

thyroid disease	N	Male (n=1358)		Female (n=1270)		χ^2^	*P*	Urban (n=1374)		Rural (n=1254)		χ^2^	*P*
		n	Percentage /%	n	Percentage /%			n	Percentage /%	n	Percentage /%		
Clinical hyperthyroidism	31	17	1.25	14	1.10	0.13	0.723	19	1.38	12	0.96	1.02	0.312
Subclinical hyperthyroidism	4	2	0.15	2	0.16	0.00	0.947	3	0.22	1	0.08	0.83	0.363
Clinical hypothyroidism	27	9	0.66	18	1.42	3.67	0.055	8	0.58	19	1.52	5.62	0.018
Subclinical hypothyroidism	768	320	23.58	448	35.28	43.40	0.000	355	25.84	413	32.96	16.01	0.000
Goiter	56	13	0.96	43	3.39	18.49	0.000	19	1.38	37	2.96	7.75	0.005
Single thyroid nodule	169	78	5.74	91	7.17	2.20	0.138	110	8.01	59	4.70	11.87	0.001
Multinodular thyroid gland	46	20	1.47	26	2.05	0.02	0.262	22	1.60	24	1.91	0.34	0.541
TPO Ab positivity	257	85	6.26	172	13.54	39.47	0.000	145	10.55	112	8.93	1.95	0.168
TG Ab positivity	242	65	4.79	177	13.94	65.73	0.000	136	9.90	106	8.45	1.64	0.201

The detection rates of subclinical hypothyroidism, and goiter were significantly higher in females (35.28%, 3.39%) than those in males (23.58%, 0.96%) (P < 0.05), and the positive rates of TPO Ab and TG Ab were significantly higher in females (13.54%, 13.94%) (P < 0.05). The detection rates of clinical hypothyroidism, subclinical hypothyroidism, and nodular goiter were significantly higher in rural populations (1.52%, 32.96%, 2.96%) than those in urban populations (0.58%, 25.84%,1.38%) (P < 0.05).

### 4.5 Thyroid diseases and iodine nutritional status

As show in **Table 6**, under different iodine nutrition statuses, there was no significant difference in the prevalence of clinical hyperthyroidism, subclinical hyperthyroidism, subclinical hypothyroidism, nodular goite, thyroid nodules detected, and positive rates of TPO Ab and TG Ab (P > 0.05). The detection rate of clinical hypothyroidism was statistically significant in different iodine nutritional states (P< 0.05).

**Table 6:** Prevalence of thyroid diseases among people with different iodine nutritional status.

thyroid disease	Deficient iodine intake(n=297)		Adequate iodine intake(n=861)		More than adequate iodine intake(n=745)		Excessive iodine intake(n=725)		χ^2^	*P*
	n	Percentage /%	n	Percentage /%	n	Percentage /%	n	Percentage /%		
Clinical hyperthyroidism	2	0.67	9	1.06	10	1.34	10	1.38	1.17	0.761
Subclinical hyperthyroidism	0	0.00	1	0.12	2	0.27	1	0.14	1.19	0.756
Clinical hypothyroidism	8	2.69	6	0.71	5	0.67	7	0.97	10.22	0.017
Subclinical hypothyroidism	81	27.27	265	30.78	203	26.57	228	29.84	3.79	0.285
Goiter	8	2.70	19	2.24	8	1.08	21	2.90	6.49	0.090
Single thyroid nodule	21	7.07	62	7.30	50	6.71	36	4.97	3.94	0.268
Multinodular thyroid gland	3	1.01	16	1.88	12	1.61	15	2.07	1.54	0.673
TPO Ab positivity	33	11.11	88	10.37	64	8.59	70	9.66	2.13	0.545
TG Ab positivity	27	9.09	79	9.31	58	7.79	76	10.48	3.24	0.357

## 5 Discussion

By synthesizing thyroid stimulating hormone (TH), iodine plays a crucial role in the growth of the body and in the development of tissue morphology. Insufficient THS production caused by iodine deficiency can lead to endemic goiter, cretinism, hypothyroidism, and even thyroid cancer (6). However, long-term excessive iodine exposure can lead to iodine induced hyperthyroidism, iodine excess goiter, chronic lymphocytic thyroiditis, and hypothyroidism (7). The Qinghai Province is situated in northwest China, on the Qinghai-Tibet Plateau. Qinghai Province is currently suffering from widespread iodine deficiency in the external environment. The implementation and continuous adjustment of USI policies have effectively controlled iodine deficiency disorder (IDD) (5). However, in the past 20 years, there have been no systematic reports on the epidemiology of thyroid diseases in our province. The iodine nutritional status is biased towards key populations in a certain region (8), specific ethnic groups in a certain region (9), or iodine deficiency disease testing (10, 11), lacking large-scale surveys. In addition, there are no reports of a correlation between iodine nutritional status and thyroid diseases.

In this study, a total of 2628 valid datas were obtained, and we found that the median UIC of the overall population was 217.90 ug/L, with a median UIC of 220.84 ug/L for males and 213.87 ug/L for females, which was higher than the national median urinary iodine of 177.89 μg/L of adults during the same period level (12). The recommended urine iodine level for normal adults in China is 100-200 ug/L. Therefore, the urine iodine level for adults in our province currently falls within the iodine excess range specified in the WHO/UNICEF/ICCIDD standard. By further grouping the age groups, the iodine intake of young and middle-aged people is relatively high, which may be related to the large dietary intake, multiple types, and excessive iodine salt intake of such people. The extent of iodine deficiency and the rate of iodine overdose are higher in rural areas. Iodine deficiency may be related to residents’ irregular iodized salt intake, such as poor economic conditions, reluctance to buy iodized salt, and purchase un-iodized salt on their own. The higher rate of excess iodine intake may be related to the high number of young people in the age group of 18-29 years old.

This study found that the detection rates of clinical hyperthyroidism, subclinical hyperthyroidism, clinical hypothyroidism, and goiter in Qinghai Province (1.20%, 0.20%, 1.00%, 2.10%) were similar to the overall national level (0.78%, 0.44%, 1.02%, 1.17%) during the same period of the TIDE study, and the positive rates of TPO Ab and TG Ab antibodies (9.80%, 9.20%) were also relatively stable compared to the TIDE study (10.19%, 9.70%). Subclinical hypothyroidism (29.20%) was higher than the national level (12.93%) during the same period, Subclinical hypothyroidism is detected at a significantly higher rate than the national average. The global reported data showed that clinical hyperthyroidism was 0.2%-1.3% (13), subclinical hyperthyroidism was 1% to 5% (14), and clinical hypothyroidism was 0.2-5.3% (13). In contrast, our data are generally consistent. Nevertheless, excessive iodine consumption in the diet may be contributing to an increase in the incidence rate of subclinical hypothyroidism in the population (15). Chronic long-term stimulation of excessive iodine may be the factor that iodine induces hypothyroidism. Furthermore, the detection rate was higher among female than among male, and in rural areas than in urban ones. However, the detection rate did not differ significantly among patients with different levels of urinary iodine.

Thyroid diseases can be caused by insufficient or excessive iodine intake, according to previous studies (16, 17). Subclinical hypothyroidism is associated with an increase in iodine intake in iodine-deficient populations. However, it has also been reported that there is no correlation between iodine nutritional status and thyroid disease. In this study, no correlation was found between iodine and subclinical hypothyroidism. Further research is needed to clarify the reference range of TSH and the correlation between iodine and thyroid function in normal populations in Qinghai Province.

According to this study, 8.2% of thyroid nodules are detected in Qinghai Province, less than 49.0% in Beijing (18), 36,88% in Heilongjiang (19), and 27.8% in Shanghai (20), and less than the overall national level of 20.43% during the same period. Female are most likely to be detected with goiter than male, and urban areas have a higher detection rate than rural areas. This result is consistent with adult domestic and foreign adult data (21, 22). There is an association between higher iodine intake and a lower prevalence of thyroid nodules in the TIDE study (3). However, we found no significant association between thyroid nodules and people with different urinary iodine levels. Therefore, the specific mechanism of thyroid nodules remains unclear. It has been reported that thyroid nodules may be related to genetics, economic development, lifestyle, and more exposure to radiation among urban populations, atmospheric and water pollution, as well as psychological stress and other predisposing factors for thyroid nodules (23, 24). More research is needed to analyze the cause.

We found that the detection rate of hypothyroidism in people with iodine deficiency is higher than in other iodine nutritional situations. Many studies have reported that low urinary iodine levels are associated with decreased thyroid function. Iodine deficiency or excessive nutrition can easily increase the prevalence of clinical and subclinical hypothyroidism (1). There were no statistical differences in the detection rates of other diseases among populations with different iodine intakes, which may be due to sampling reasons or other learning disturbances, etc.

This study is the first epidemiological investigation to comprehensively evaluate iodine nutrition and thyroid diseases in Qinghai Province. We found that the iodine nutrition level in Qinghai Province is currently in excess of iodine. The prevalence of most thyroid diseases remained stable, with a paradoxical significant decrease in the rate of thyroid nodule detection and a significant increase in the proportion of subclinical hypothyroidism. It should continue to be implemented and monitored, while actively searching for the causes of subclinical hypothyroidism and taking the corresponding countermeasures. The incidence rate of thyroid nodules is low, but the detection of malignancy should be done well. The inadequacies of this article are due to the fact that only two residential communities in urban and rural areas were selected, and the samples couldn’t represent the entire population. Furthermore, Qinghai province has a different high altitudes, and whether there are differences between different altitudes requires further research.

## Data availability statement

The raw data supporting the conclusions of this article will be made available by the authors, without undue reservation.

## Ethics statement

The research protocols were approved by the Medical Ethics Committee of China Medical University. All subjects were provided written informed consent following a thorough explanation of the research procedures. Some measures were taken to ensure the project quality. The studies were conducted in accordance with the local legislation and institutional requirements. Written informed consent for participation in this study was provided by the participants’ legal guardians/next of kin.

## Author contributions

XF, LZ, and CC collect data and write the article. All authors contributed to the article and approved the submitted version.
